# Coded Cooperation for Multiway Relaying in Wireless Sensor Networks ^†^

**DOI:** 10.3390/s150715265

**Published:** 2015-06-29

**Authors:** Zhongwei Si, Junyang Ma, Ragnar Thobaben

**Affiliations:** 1 Key Lab of Universal Wireless Communications, Ministry of Education, Beijing University of Posts and Telecommunications(BUPT), 100876 Beijing, China; E-Mail: majunyang@bupt.edu.cn; 2 School of Electrical Engineering and ACCESS Linnaeus Center, KTH Royal Institute of Technology, 10044 Stockholm, Sweden; E-Mail: ragnart@kth.se

**Keywords:** wireless sensor networks, multiway relay channel, capacity region, coset encoding, multi-edge-type LDPC codes, spatially-coupled LDPC codes

## Abstract

Wireless sensor networks have been considered as an enabling technology for constructing smart cities. One important feature of wireless sensor networks is that the sensor nodes collaborate in some manner for communications. In this manuscript, we focus on the model of multiway relaying with full data exchange where each user wants to transmit and receive data to and from all other users in the network. We derive the capacity region for this specific model and propose a coding strategy through coset encoding. To obtain good performance with practical codes, we choose spatially-coupled LDPC (SC-LDPC) codes for the coded cooperation. In particular, for the message broadcasting from the relay, we construct multi-edge-type (MET) SC-LDPC codes by repeatedly applying coset encoding. Due to the capacity-achieving property of the SC-LDPC codes, we prove that the capacity region can theoretically be achieved by the proposed MET SC-LDPC codes. Numerical results with finite node degrees are provided, which show that the achievable rates approach the boundary of the capacity region in both binary erasure channels and additive white Gaussian channels.

## 1. Introduction

Wireless sensor networks (WSNs) consist of spatially-distributed autonomous sensors which cooperatively monitor physical or environmental conditions, and they have been identified as one of the most important enabling technologies for constructing smart cities. The rapid development of portable and wearable devices has further fueled the application of sensor networks, which helps to improve the city's sustainable growth and citizens' living quality.

One important feature of WSNs is that the sensor nodes collaborate in some manner for information transmissions. Much of research on the physical-layer techniques has focused on relaying, which is an efficient cooperative strategy to improve throughput and coverage of the sensor network. The three-node relay channel [1], consisting of one source, one relay and one destination, serves as the fundamental unit of large networks. The two-way relaying, also known as bidirectional relaying [2], is another type of cooperative strategy. By exploiting bidirectional communication, the loss in spectral efficiency due to the half-duplex restriction in conventional relaying is reduced. As a generalization of the two-way relay channel, the multiway relay channel (mRC) has been investigated in [3]. In the mRC, multiple users exchange information with the help of a relay terminal. The mRC setup models a large variety of communication scenarios. For example, in a physical sensor network, temperature sensors exchange local temperature measurements among themselves. Similarly, in a social network, different users exchange their personal information through a coordinator.

In this manuscript, we are interested in a typical scenario which is called the multiway relay channel with full data exchange in [3] or the conferencing multiway channel in [4]. A practical example of this model is as follows. Each member of an emergency response team is equipped with a wireless device at a disaster site, and they may obtain valuable information at random times. All of the members, including one leader, need to share their messages with the others. Each member wants to transmit and receive data to and from all other responders, coordinated by the leader who acts as the relay. The above information exchange can be realized in two steps. First, since the members may initiate their transmissions asynchronously, each member is scheduled to transmit his or her message to the leader in a time-division manner. During this procedure, the message in the air may be overheard by other members. After successfully receiving all of the messages, the leader (relay) then encodes all of these messages together with his or her message and broadcasts the resulting codeword to all of the members. At each member's side, different and potentially partly overlapping *a priori* information is available for decoding the codeword from the relay. Eventually, all of the members in the team are supposed to obtain the messages from all of the others. Since the information delivery in the first phase is essentially a peer-to-peer communication, in this manuscript, we focus on the second phase in which the relay broadcasts the messages to all of the members.

The capacity region for the general multi-user broadcast channel is not known yet. In [5,6], the authors provided the capacity region for the bidirectional broadcast channel with a common and/or private message. The multiway relay channel with full data exchange as we described above can be considered as a generalization of the model in [6]. In this manuscript, we derive the capacity region for the multiway relay case. The capacity region establishes the fundamental limits of the transmission rate and provides guidelines for the code design. On top of this, we propose a coding strategy through coset encoding for broadcasting with receiver side information. The coding strategy is illustrated by using low-density parity-check codes [7]. However, in practice, the regular LDPC codes cannot provide satisfactory performance, while irregular LDPC codes suffer from the complicated optimization of the degree distribution [8]. Therefore, it is of interest to search for code constructions with a good performance and a low optimization overhead. Different designs can be found for various channel models in the literature based on Turbo codes [9,10], LDPC codes [11-13], Polar codes [14], *etc.*

The spatially-coupled LDPC (SC-LDPC) codes are good candidate for the cooperative code construction. Spatially-coupled LDPC codes were first introduced in [15] as a time-varying convolutional-like LDPC code family. Then, the idea was further developed in, e.g., [16-18], where different constructions and analytical results were provided. It has been proven analytically in [18,19] that the belief-propagation (BP) decoding threshold of a spatially-coupled LDPC code ensemble achieves the maximum *a posteriori* probability (MAP) threshold of the underlying LDPC block code. This code, in turn, approaches capacity as the node degrees increase. In addition to the capacity-achieving performance for various channels, the regularity of the SC-LDPC code allows us to avoid complicated re-optimization of the degree distributions for varying channel conditions. Meanwhile, this code enables recursive encoding and sliding-window decoding [20], which dispels the concerns about hardware complexity and delay. Motivated by these useful properties, spatially-coupled LDPC codes have been considered for applications in a variety of scenarios.

In this manuscript, we propose to use spatially-coupled LDPC codes for the cooperative channel coding in the mRC. To realize the proposed coding strategy by using SC-LDPC codes, we replace the LDPC codes with their spatially-coupled counterparts. The multi-edge-type (MET) [21] nested spatially-coupled LDPC codes are constructed by repeatedly applying the coset encoding [22]. If setting the node degree the same for all types of check nodes, the MET nested SC-LDPC codes present useful properties. Any combination of the nesting leads to a capacity-achieving SC-LDPC code, regardless of the number of edge types and the order of adding the edges. This property is beneficial for the model under discussion. We then prove that by using the MET nested SC-LDPC code, the capacity region of the channel model is achieved. Numerical results with finite node degrees are provided, which show that the achievable rates approach the boundary of the capacity region in both binary erasure channels (BECs) and binary-input additive white Gaussian channels (BI-AWGNs).

The remainder of the manuscript is organized as follows. The system model is introduced in Section 2. In Section 3, we derive the capacity region of the specific model. The coding strategy is illustrated in Section 4 through multi-edge-type LDPC codes. Based on the multi-edge-type construction, the MET spatially-coupled LDPC codes are proposed in Section 5. In Section 6, we apply the MET SC-LDPC codes to the multiway relay channel and prove that the capacity region can be achieved. The numerical results are provided in Section 7 to verify the theoretical analysis. Finally, Section 8 concludes the manuscript.

## 2. System Model

### 2.1. The Two-Way Relay Channel

**Figure 1:**
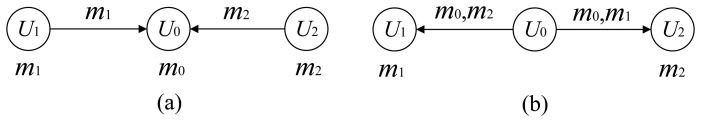
The two-way relay channel with a common message. (**a**) The users transmit to the relay in turn; (**b**) The relay broadcasts to the users.

We first use a special case, the two-way relay channel, to illustrate the system model. We assume that there are three users *U*_0_, *U*_1_ and *U*_2_ in the network, and they have individual messages ***m***_0_, ***m***_1_ and ***m***_2_ to share with the others. Without loss of generality, we choose user *U*_0_ to act as the relay. In the first phase, the users *U*_1_ and *U*_2_ transmit their messages ***m***_1_ and ***m***_2_, respectively, in turn to the relay node *U*_0_. We assume that both messages are received successfully by *U*_0_. In the second phase, *i.e.*, the broadcast phase, the relay *U*_0_ transmits a codeword to both *U*_1_ and *U*_2_. The goal of the transmission from *U*_0_ is to convey the messages ***m***_0_, ***m***_1_ and ***m***_2_ efficiently, so that *U*_1_ is able to reliably decode the messages ***m***_0_ and ***m***_2_ and *U*_2_ is able to reliably decode the messages ***m***_0_ and ***m***_1_. Eventually, all three users share the three messages in the network. The channel model is illustrated in Figure 1. This model is recognized as the two-way relay channel with a common message in [6].

### 2.2. The Multiway Relay Channel with Full Data Exchange

In the following, we extend the model to the multiway relay channel with full data exchange. We assume that a number of *K* users {*U*_0_, *U*_1_, …,*U_l_*, … *U_k_*_– 1_ } form a network. Each user *U_l_* has an individual message ***m****_l_* to share with the others, for 0 ≤ l ≤ *K* ‒ 1. One of the users, for example *U*_0_, is assigned as the relay node. In the first phase, all of the users, except the relay *U*_0_, send out their messages in a time-division manner. During this procedure, each message ***m****_l_*, 1 ≤ l ≤ *K* – 1, is successfully received by the relay and may be received by other users. In the second phase, the relay *U_0_* has the task of broadcasting a set of messages 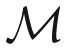 = {***m***_1_, ***m***_2_,…, ***m****_k_*_–1_} which he has received beforehand, together with his own message ***m***_0_, to all of the users. The relay combines the messages 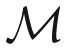 and ***m***_0_ and encodes them into a codeword ***X***. At each user *U_l_*, a non-empty subset of 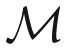, which includes at least his own message ***m****_l_*, is available as the side information for decoding. For a subgroup of the users, the side information may be: (i) non-overlapping; (ii) partly overlapping; or (iii) completely overlapping. We assume that the relay node has the knowledge of what side information is available at each user and of the channel condition between the relay and each user. The broadcasting from the relay should be dedicated to transmit all of the messages efficiently, considering the available information.

**Figure 2:**
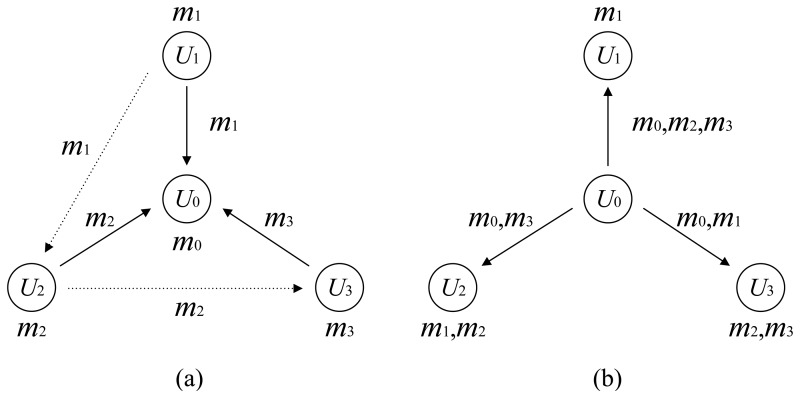
One example of the multiway relay channel with full data exchange. (**a**) The users transmits to the relay in turn, and the messages may be overheard by other users; (**b**) The relay broadcasts to all the users.

In Figure 2, we give a simple example of the above multiway relay channel. The example system consists of four users, and one of them is selected as the relay. In the first round of transmission, the message ***m***_1_ from *U*_1_ is successfully received by both the relay and the user *U*_2_. The message ***m***_2_ from *U*_2_ is successfully received by the relay and the user *U*_3_. The message ***m***_3_ is only correctly received at the relay Each user can use the messages he has overheard together with his own message as the side information for decoding. That is, *U*_1_ has only his own message ***m***_1_ as the side information. *U*_2_ has ***m***_1_ and ***m***_2_ as the *a priori* information, and *U*_3_ has ***m****_2_* and ***m***_3_ as the *a priori* information for the decoding. In the end of the first phase, each user informs the relay about what side information he has. This can be realized at each user by sending the indexes of his received messages to the relay. The number of bits needed to represent the index of one message is ⌈log_2_(*K* − 1)⌉, where *K* is the number of users. The total number of bits for each user to send the knowledge of available information is at most (*K* ‒ 2) ⌈log_2_(*K* − 1)⌉. For the example in Figure 2, each user only needs four bits. Comparing to the length of the messages, the resulting overhead and latency are negligible. In the broadcast phase, the relay delivers the missing messages to each user, *i.e.*, {***m***_0_,***m***_2_,***m***_3_} to *U*_1_, {***m***_0_,***m***_3_} to *U*_2_ and {***m***_0_,***m***_1_} to *U*_3_. The challenge for the relay is then how to realize the information broadcasting efficiently and reliably.

## 3. Capacity Region

The capacity region for the two-way relay channel with a common message has been proved in [6]. Let *R*_0_, *R*_1_ and *R*_2_ be the encoding rates of the messages ***m***_0_, ***m***_1_ and ***m***_2_, and let the capacities of the links to *U*_1_ and *U*_2_ be *C*_1_ and *C*_2_, respectively. The capacity region of the above model is then given by the set of triples (*R*_0_, *R*_1_, *R*_2_) that satisfies [6,14]:

$$\begin{array}{c} R_{0}+R_{2}\leq C_{1}\\ R_{0}+R_{1}\leq C_{2} \end{array}$$

provided that *R*_0_ ≤ min (*C*_1_, *C*_2_).

In the multiway broadcast channel that we described in Section 2.2, each user requests all of the messages he has missed from 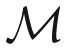 plus the message ***m***_0_ from the relay. We assume that the receiver side information available at *U_l_* is a set 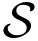
*_l_* , ***m****_l_* ⊆ 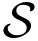
*_l_* ⊆ 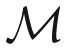. Then, the messages that should be delivered from the relay to *U_t_* can be denoted by 
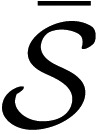
 ∪ ***m***_0_. This assumption simplifies the general problem of multi-user broadcasting, and therefore, the capacity region of the model can be obtained. We give the corresponding capacity region in the following theorem.

**Theorem 1.** *We assume that C_l_ is the channel capacity of the link between the relay and the user U_l_, for all l* ∈ {1, 2,…, K – 1}. *We let R_l_ denote the encoding rate of the message m_l_ and R*_0_ *denote the encoding rate of the message m*_0_ *from the relay. The capacity region of the multiway relay channel with full data exchange is the set of all achievable rates that satisfies:*

$$\begin{array}{c} R_{0}+\underset{i:m_{i}\in {\bar{S}}_{1}}{\text{∑}}R_{i}\leq C_{1}\\ R_{0}+\underset{i:m_{i}\in {\bar{S}}_{2}}{\text{∑}}R_{i}\leq C_{2}\\ \ldots \\ \begin{array}{c} \begin{array}{c} R_{0}+\underset{i:m_{i}\in {\bar{S}}_{1}}{\text{∑}}R_{i}\leq C_{l}\\ \ldots \end{array}\\ R_{0}+\underset{i:m_{i}\in {\bar{S}}_{k-1}}{\text{∑}}R_{i}\leq C_{k-1} \end{array} \end{array}$$

*provided that: (i) R*_0_ ≤ min(*C*_1_, *C*_2_,…, *C*_k–1_)*; and (ii) C_l_* ≥ *C*_k_ if 
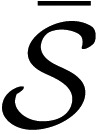
 ⊇ 
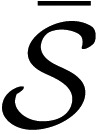
, *for any l, k* ∈ {1, 2, …,*K*−1}.

**Proof.** Without loss of generality, we use the link between the relay and the user *U_l_* (1 ≤ l ≤ *K* ‒ 1) to discuss the converse and the achievability of the capacity region. We assume that the messages are encoded by a length-*n* codeword ***X***, and the channel observation at *U_l_* is ***Y****_l_*.

First, the entropy can be bounded similarly as has been done in [6], where a two-user case was considered.

$$\begin{array}{l} \;H\left(M_{0},{\bar{S}}_{l}\right)\\ \overset{\left(a\right)}{=}H\left(M_{0},{\bar{S}}_{l}|S_{l}\right)\\ \overset{\left(b\right)}{=}I\left(M_{0},{\bar{S}}_{l};Y_{l}|S_{l}\right)+H\left(M_{0},{\bar{S}}_{l}|Y_{l},S_{l}\right)\\ \overset{\left(c\right)}{\leq }I\left(M_{0},{\bar{S}}_{l};Y_{l}|S_{l}\right)+n\varepsilon_{l}\\ \overset{\left(d\right)}{\leq }I\left(M_{0},{\bar{S}}_{l},S_{l};Y_{l}\right)+n\varepsilon_{l}\\ \overset{\left(e\right)}{\leq }I\left(X;Y_{l}\right)+n\varepsilon_{l}. \end{array}$$

Equations (a) and (b) are obtained from the independence of the messages and the definition of mutual information. Inequation (c) has been proven in [6]. Inequations (d) and (e) are based on the properties of mutual information.

Dividing both sides by *n* and using the memoryless property, we get:

$$\frac{1}{n}H(M_{0},{\bar{S}}_{l})\leq \frac{1}{n}\sum_{i=1}^{n}I(X_{i},Y_{l_{i}})+\varepsilon_{l},$$

where the intermediate steps are omitted.

Since *ε_l_* → 0 when *n* → ∞, we have the achievable rate on the relay-*U_l_* link:

$$R_{0}+\sum_{i:m_{i}\in {\bar{S}}_{l}}R_{i}\leq C_{l}.$$

Similarly, we obtain the rates for other relay-user links as in Theorem 1. Note that the conditions (i) and (ii) guarantee the nonnegativity of the rates.

The above proves the converse of the capacity region. The achievability of the capacity region can be done similarly as for the two-user case. Since we will prove in the remainder of the manuscript that the capacity region can be achieved by using the proposed code structure, we omit the proof of achievability here.

For the example in Figure 2, the capacity region is given as:

$$\begin{array}{r} R_{0}+R_{2}+R_{3}\leq C_{1}\\ R_{0}+R_{3}\leq C_{2}\\ R_{0}+R_{1}\leq C_{3} \end{array}$$

Note that this enforces *R*_0_ ≤ min(*C*_1_, *C*_2,_*C*_3_) and *C*_2_ ≤ *C*_1_.

## 4. Multi-Edge-Type LDPC Codes and the Coding Strategy

### 4.1. Low-Density Parity-Check Codes

As the name suggests, LDPC codes are block codes with parity-check matrices which contain only a small proportion of non-zero entries. An LDPC code can be represented by a parity-check matrix or a Tanner graph.

A parity-check matrix ***H*** with elements in GF(2) defines the codeword constraints in a matrix form. For a binary code with *k* parity-check equations and with codeword length *n*, the matrix ***H*** is of size *k* × *n*. A vector ***X*** = [*X*_1_, *X*_2_, …, *X_n_*] is a codeword of the LDPC code defined by ***H*** if and only if the following constraint is satisfied:

$${\text{HX}}^{T}=0$$

In the following, we use 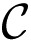 to represent a code and use ***X*** to denote one codeword of 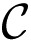.

Tanner introduced an effective graphical representation for LDPC codes [23]. In a bipartite Tanner graph, the nodes are separated into two distinctive sets: variable nodes for the codeword bits and check nodes for the parity-check equations. An edge connects a variable node to a check node if that codeword bit is included in the parity-check equation.

As originally defined by Gallager, a (*d_v_*, *d_c_*) regular LDPC code is determined by the condition that every codeword bit participates in exactly *d_v_* parity-check equations and that every parity-check equation involves exactly *d_c_* codeword bits. The parameter *d_v_* is called the variable node degree, and *d_c_* is the check node degree.

Since each parity-check equation typically reduces the number of degrees of freedom by one, it follows that the design rate of the (*d_v_*, *d_c_*) regular LDPC code is:

(1) $$R(d_{v},d_{c})=\frac{n-k}{n}=1-\frac{d_{v}}{d_{c}}$$

### 4.2. Binning and Coset Coding

One of the basic elements of network information theory is the idea of binning [24]. A binning scheme divides a set of codewords into subsets (“bins”), such that the codewords in each subset are as far apart as possible. In [24], random binning was used to prove the Slepian-Wolf source coding theorem. In [25], Cover and El Gamal proposed to use random binning for set partitioning in decode-and-forward relaying.

The random binning approach is convenient for theoretical analysis; however, it is not suitable for practical applications. Therefore, binning schemes with a structure were proposed. Wyner constructed an algebraic binning scheme for noiseless coding problems in [26]. In order to extend the idea to “noisy” coding problems, the structure of nested codes was proposed [27].

We first introduce the concept of coset. Let an LDPC code 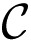 be specified by a *k* × *n* binary parity-check matrix ***H***. The code 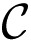 = {***X*** : ***HX****^T^* = 0} contains all length-*n* binary vectors whose syndrome ***S*** = ***HX****^T^* is equal to 0. Given some general syndrome ***S*** ∈ {0, 1}*^k^*, the set of all length-*n* binary vectors ***X*** satisfying ***HX****^T^* = ***S*** is called a coset 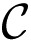
*_S_*.

A nested code [27] is a pair of linear codes ( 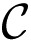
_1_, 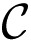
_2_) that satisfies:

$$C_{2}\subset C_{1}$$

That is, each codeword of 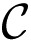
_2_ is also a codeword of 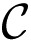
_1_.

The nested parity-check codes can be constructed as follows [27]. Let ***H***_1_ and ***H***_2_ be parity-check matrices of dimension *k*_1_×*n* and of dimension *k*_2_ × *n*, respectively, *k*_2_ > *k*_1_. By bringing in an additional parity-check matrix **Δ*H*** of dimension (*k*_2_ – *k*_1_) × *n*, the nested codes can be realized by:

$$H_{2}=\left[\begin{array}{c} H_{1}\\ \Delta H \end{array}\right]$$

We can then partition 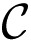
_1_ into 2*^k^*^_2_^^−^*^k^*^_1_^ cosets of 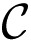
_2_.

### 4.3. MET Nested LDPC Codes

Multi-edge-type LDPC codes [21] can be considered as a generalization of regular and irregular LDPC codes. For an MET LDPC ensemble, there are multiple equivalence classes of edges, while for a conventional LDPC ensemble, there exists a single type of edge. In the following, we introduce multi-edge-type LDPC codes constructed by coset encoding.

**Figure 3:**
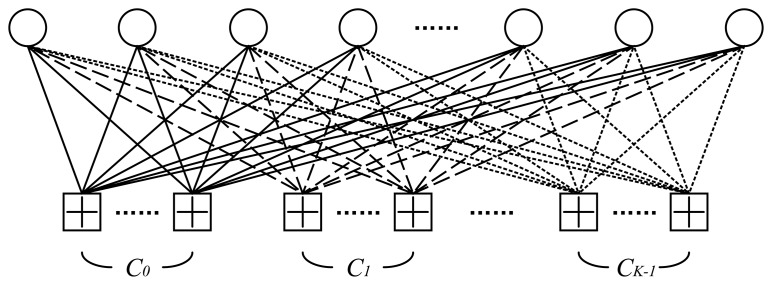
Multi-edge-type LDPC codes.

An MET nested LDPC code can be illustrated by the Tanner graph in Figure 3. The bit vector associated with the variable nodes ***V***, *i.e.*, the codeword, is denoted by ***X***. There are *K* types of check nodes in the graph, ***C***_0_, ***C***_1_, …, ***C****_l_* …, ***C****_k_*_−1_. The parity-check matrices, which correspond to the *K* types of edges connecting the variable nodes and the different types of check nodes, are denoted by ***H***_0_, ***H***_1_, …, ***H****_l_*, …, ***H****_k-_*_1_, and have variable and check node degrees (*d_v_*__0__, *d_c_*__0__), (*d_v_*__1__, *d_c_*__1__), …, (*d_v_l__*, *d_c_l__*), …, (*d_v_*_*k*_−1__, *d_c_*_*k*_−1__), respectively. The MET nested LDPC code can be described by the stacked parity-check matrix ***H***, and we have:

(2) $${\text{HX}}^{T}=\left[\begin{array}{c} H_{0}\\ H_{1}\\ \vdots \\ H_{K-1} \end{array}\right]\;X^{T}=0$$

The definition of a multi-edge-type nested LDPC code ensemble is given in the following.

**Definition 2.** (*The multi-edge-type nested LDPC code ensemble) A multi-edge-type nested LDPC ensemble includes all of the bipartite graphs where each variable node is connected to d_vl_ type-l check nodes, and each of the type-l check nodes has d_Cl_ edges connecting to the variable nodes, for all l* ∈ [0, *K* − 1].

### 4.4. Coding Strategy for Multiway Relaying

Based on the nested LDPC codes, in the following, we illustrate the coding strategy for the multiway relay channel with full data exchange. Since the first phase is in fact a peer-to-peer transmission, we focus particularly on the transmission strategy in the broadcast phase. We start with the case of two users and one relay. We assume that the binary messages ***m***_0_, ***m***_1_, and ***m***_2_ are of lengths *k*_0_, *k*_1_ and *k*_2_ bits. To combine the three messages in a single transmission, the relay uses a double binning strategy: a high-rate code of length *n* is split into 2*^nR^*^1^ × 2*^nR^*^2^ disjoint sub-codes 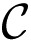
*_i_*,*_j_*, with *i* ∈ {1,…,2 *^nR^*^1^} and *j* ∈ {1,…, 2*^nR^*^2^}, each of rate *R*_0_. Note that *k*_0_ = *nR*_0_, *k*_1_ = *nR*_1_ and *k*_2_ = *nR*_2_. For encoding, the relay lets the *k*_1_ and *k*_2_ message bits ***m***_1_ and ***m***_2_ determine which sub-code is selected, and he uses the chosen sub-code 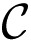 *_m_*__1_,_*_m_*__2__ for encoding ***m***_0_. This encoding strategy can be realized by using a coset code 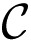
*_m_*__1_,_*_m_*__2__ of the MET nested LDPC code that is selected by the messages ***m***_1_ and ***m***_2_ as follows:

(3) $$\left[\begin{array}{c} H_{0}\\ H_{1}\\ H_{2} \end{array}\right]\;X^{T}=\left[\begin{array}{c} 0\\ m_{1}\\ m_{2} \end{array}\right]$$

where ***H***_0_ is a (*k′* × *n*) matrix, *k′* satisfies *n* = *k*_0_ + *k′* + *k*_1_ + *k*_2_, ***H***_1_ is a (*k*_1_ × *n*) matrix and ***H***_2_ is a (*k*_2_ × *n*) matrix.

Once the Tanner graph of an MET nested LDPC code is given or, equivalently, the parity-check matrices ***H***_0_, ***H***_1_, and ***H***_2_ are given, the encoding can be carried out by mapping the message ***m***_0_ into a codeword which simultaneously satisfies the check constraints (3).

If *d_C_*__0__ = *d_C_*__1__ = *d_C_*__2__ ≡ *d_c_*, the rate for encoding ***m***_0_ is:

(4) $$R_{0}=\frac{k_{0}}{n}=1-\frac{k^{\prime }+k_{1}+k_{2}}{n}=1-\frac{d_{v_{0}}+d_{v_{1}}+d_{v_{2}}}{d_{c}}$$

and the rates for encoding ***m***_1_ and ***m***_2_ are, respectively:

(5) $$R_{1}=\frac{k_{1}}{n}=\frac{d_{v_{1}}}{d_{c}}$$

and:

(6) $$R_{2}=\frac{k_{2}}{n}=\frac{d_{v_{2}}}{d_{c}}$$

The decoding at each user is carried out as follows. Since *U*_1_ has perfect knowledge of ***m***_1_, he interprets ***X*** as a codeword of a two-edge-type nested LDPC code and uses:

$$\left[\begin{array}{c} H_{0}\\ H_{1} \end{array}\right]\;X^{T}=\left[\begin{array}{c} 0\\ {\text{m}}_{1} \end{array}\right]$$

to decode the codeword ***X***. With the decoded codeword ***X̂***, *U*_1_ obtains ***m***_0_ and recovers ***m***_2_ by using the fact that ***m***_2_ = ***H***_2_***X****^T^* following from Equation (3). In a similar way, *U*_2_ recovers the messages ***m***_0_ and ***m***_1_ by using ***m***_2_ as side information.

The above coding strategy can be easily extended to the multi-user case. A multiple binning technique is utilized to combine all of the messages in a single transmission. For the general case, the encoding can be realized by selecting a codeword ***X*** that satisfies:

(7) $$\left[\begin{array}{c} H_{0}\\ H_{1}\\ H_{2}\\ \vdots \\ H_{K-1} \end{array}\right]\;X^{T}=\left[\begin{array}{c} 0\\ m_{1}\\ m_{2}\\ \vdots \\ m_{K-1} \end{array}\right]$$

By choosing *d_C_l__* = *d_c_* for all *l* ∈ {0,1, 2,…, *K* – 1}, we get the rate for encoding ***m***_0_ as:

$$R_{0}=1-\frac{\sum_{l=0}^{K-1}d_{v_{l}}}{d_{c}}$$

The rate for encoding the message ***m****_l_* is:

$$R_{l}=\frac{d_{v_{l}}}{d_{c}}$$

for *l* ∈ {1,2,…,*K*−1}.

When decoding, each user utilizes the side information available at the receiver. We assume that the indexes of the available messages at *U_l_* form a vector ***v**_l_* of length *P_l_*. Then, the decoding of the codeword ***X*** (the message ***m***_0_) at *U_l_* is carried out by satisfying:

(8) $$\left[\begin{array}{c} H_{0}\\ H_{v_{l}(1)}\\ H_{v_{l}(2)}\\ \vdots \\ H_{v_{l}(P_{l})} \end{array}\right]\;X^{T}=\left[\begin{array}{c} 0\\ m_{v_{l}(1)}\\ m_{v_{l}(2)}\\ \vdots \\ m_{v_{l}(p_{l})} \end{array}\right]$$

As for the two-user case, the other messages designated to *U_l_* can be recovered by using the decoded ***X*** and sub-parity-check equations in Equation (7).

We again have a look at the example in Figure 2. The encoding can be realized by selecting a codeword ***X*** that satisfies that:

$$\left[\begin{array}{c} H_{0}\\ H_{1}\\ H_{2}\\ H_{3} \end{array}\right]\;X^{T}=\left[\begin{array}{c} 0\\ m_{1}\\ m_{2}\\ m_{3} \end{array}\right]$$

The decoding is carried out by satisfying:

(9a) $$\left[\begin{array}{c} H_{0}\\ H_{1} \end{array}\right]\;X^{T}=\left[\begin{array}{c} 0\\ m_{1} \end{array}\right]$$

at *U*_1_, satisfying:

(9b) $$\left[\begin{array}{c} H_{0}\\ H_{1}\\ H_{2} \end{array}\right]\;X^{T}=\left[\begin{array}{c} 0\\ m_{1}\\ m_{2} \end{array}\right]$$

at *U*_2_ and satisfying:

(9c) $$\left[\begin{array}{c} H_{0}\\ H_{2}\\ H_{3} \end{array}\right]\;X^{T}=\left[\begin{array}{c} 0\\ m_{2}\\ m_{3} \end{array}\right]$$

at *U*_3_.

In the above, we have illustrated the coding strategy using LDPC codes. In general, the regular LDPC codes suffer from poor performance. The performance can be improved by introducing irregularity to the node degrees. However, an irregular degree distribution needs to be derived to match a given channel condition, which complicates the code design. Therefore, we are interested in searching for code constructions with a good performance and a low optimization overhead. The spatially-coupled LDPC codes are then good candidates for this purpose.

## 5. Multi-Edge-Type Spatially-Coupled LDPC Codes

In this section, we first give a brief introduction of spatially-coupled LDPC codes. Then, we introduce the construction of multi-edge-type nested SC-LDPC codes. The property of the proposed codes is analyzed. We will show that any *N*-edge-type nested SC-LDPC code embedded in the MET structure is capacity achieving.

### 5.1. Spatially-Coupled LDPC Codes

A regular {*d_v_*,*d_c_*} binary spatially-coupled LDPC code can be defined by an infinite parity-check matrix: [17]

$$H_{\infty }=\left[\begin{array}{ccccc} \ddots & & & & \\ & h_{0}(1) & & & \\ \ddots & \vdots & \ddots & & \\ & h_{w-1}(1) & & h_{0}(t) & \\ & & \ddots & \vdots & \ddots \\ & & & h_{w-1}(t) & \\ & & & & \ddots \end{array}\right]$$

where *d_v_* is the variable node degree and *d_c_* is the check node degree. We assume that at each position *t* (*t* ∈ (−∞, ∞)), the number of variable nodes is *M*, and accordingly, the number of check nodes is *Md_v_*/*d_c_*. Then, each submatrix ***h****_i_*(*t*) is an (*Md_v_*/*d_c_*) × *M* binary matrix. In practice, for a finite number of positions *L*, we have t ∈ [1, *L*], and the code needs to be initialized in the beginning and terminated in the end. All of the submatrices ***h****_i_*(*t*) are sparse, and accordingly, the overall parity-check matrix is sparse.

There are many variations of spatially-coupled LDPC codes in the literature [15-18]. In this manuscript, we consider a generalized type of SC-LDPC code, which can be denoted by five parameters {*d_v_*, *d_c_*, *M*, *L*, *w*} [18]. We assume that each of the *d_v_* edges of a variable node at position *t* uniformly and independently connects to the check nodes in the range [*t*, *t* + *w* − 1], where the parameter *w* is a positive integer. Ignoring the boundary effects, there are in total a number of *Md_v_*/*w* edges coming from all of the variable nodes at position *t* − *τ*, *τ* ∈ [0, *w* − 1], to all of the check nodes at position *t*. Distributing these *Md_v_*/*w* edges uniformly at random to the *Md_v_*/*d_c_* check nodes at position *t*, for each check node, each edge is connected to the variable nodes at position *t* − *τ* with probability 1/*w*. Therefore, each of the *d_c_* connections of a check node at position *t* is considered to be uniformly and independently chosen from the range [*t* − *w* + 1, t].

It has been shown analytically in [18] that, for transmission over the BEC, the BP decoding threshold of an SC-LDPC code ensemble {*d_v_*, *d_c_*, *M*, *L*, *w*} converges to the MAP threshold of its underlying component ensemble {*d_v_*, *d_c_*} in the limit of large *M*, *L* and *w*. In the remainder of this manuscript, we will use the following properties, which were derived in [18] for the BEC case: for the design rate of the code, *R*, we have:

(10) $$\underset{w\to \infty }{\lim}\underset{L\to \infty }{\lim}\underset{M\to \infty }{\lim}R(d_{v},d_{c},M,L,w)=1-\frac{d_{v}}{d_{c}}$$

and for the BP decoding threshold, *ϵ^BP^*, we have:

(11) $$\underset{w\to \infty }{\lim}\underset{L\to \infty }{\lim}\underset{M\to \infty }{\lim}\epsilon^{BP}(d_{v},d_{c},M,L,w)=\epsilon^{MAP}(d_{v},d_{c})$$

where *ϵ^MAP^* is the MAP threshold of its block code counterpart.

For a given rate *R*, the Shannon limit is defined as the ultimate channel parameter threshold, below which reliable communication can be achieved by using optimal codes and optimal decoding. For the BEC, the Shannon limit is *ϵ^sh^* = 1 − *R*. If we increase the node degrees *d_v_* and *d_c_* while keeping the ratio λ = *d_c_*/*d_v_* fixed, *i.e.*, keeping the rate of the code fixed, the MAP threshold in Equation (11) approaches the Shannon limit,

(12) $$\underset{d_{v}\to \infty }{\lim}\epsilon^{\text{MAP}}(d_{v},d_{c}=\lambda d_{v})=\frac{1}{\lambda }=1-R=\epsilon^{Sh}$$

In this sense, the SC-LDPC code ensemble is capacity achieving for the BEC. This result was generalized in [19], where it is concluded that the SC-LDPC code ensemble universally achieves capacity over binary memoryless symmetric (BMS) channels. More details of code construction and theoretical analysis can be found in [18,19] and the references therein.

### 5.2. MET Nested SC-LDPC Codes

Based on the standard SC-LDPC code ensemble, we construct the MET nested SC-LDPC code as follows. We assume the number of variable nodes at each position to be *M*; then, for a code with *L* positions, the codeword length is *N_v_* = *ML*. The connections between the variable nodes and the *K* types of check nodes are characterized by {*d_Vl_*, *d_Cl_*, *M*, *L*, *w*}, for *l* ∈ [0, *K* − 1]. We set the smoothing parameter *w* to be the same for all of the connections. Note that only edges of the same type are connected to one check node. The connections between the variable nodes and any two types of check nodes construct a two-edge-type SC-LDPC code embedded in the MET nested code. We denote the two-edge-type SC-LDPC code with the ***m***-th and the *n*-th check nodes by 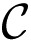
_{_*_m_*_,_*_n_*_}_ = {*d_v_*_{_*_m_*_,_*_n_*_}_,*d_c_*_{_*_m_*_,_*_n_*_}_, *M*, *L*, *w*}, ***m*** ∈ [0, *K* − 1], *n* ∈ [0, *K* − 1] and ***m*** ≠ *n*. The variable node degrees are *d_V__m_* and *d_Vn_*, and the check node degrees are *d_C__m_* and *d_Cn_*. In a similar manner, we construct the N-edge-type (*N* ∈ [1, *K*]) nested SC-LDPC code as follows. We define a set Φ of cardinality *N*, whose elements are the indices of the types of edges/check nodes chosen by the N-edge-type graph. We denote the N-edge-type code by 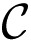
_Φ_ = {*d_vΦ_*, *d_cΦ_*, *M*, *L*, *w*}. The corresponding node degrees are {(*d_vl_*, *d_cl_*) : *l* ∈ Φ}. The general definition of the N-edge-type SC-LDPC ensemble is given as follows.

**Definition 3.** (*The N-edge-type SC-LDPC code ensemble*) *For a set* Φ ⊆ [1, *K*] *of cardinality N, N* ∈ [1, *K*], *the MET nested SC-LDPC code ensemble* 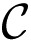
_Φ_ = {*d*_v_Φ__, *d*_c_Φ__, *M*, *L*, *w*} *of codeword length M L includes all of the bipartite graphs where each variable node is connected to d_vl_ type-l check nodes, and each type-l check node has d_cl_ edges connecting to the variable nodes, for all l* ∈ Φ. *More specifically, each of the d_vl_ type-l edges of a variable node at position t uniformly and independently connects to the type-l check nodes in the range* [*t*,*t* + *w* − 1].

### 5.3. Properties of MET SC-LDPC Codes

In this section, we discuss the performance of the MET nested SC-LDPC codes.

We have shown in [22] that, when choosing the same check degree for both types of connections, the two-edge-type nested SC-LDPC code is equivalent to a standard SC-LDPC code in terms of design rate and BP decoding threshold. Therefore, the two-edge-type nested SC-LDPC code is able to provide capacity-achieving performance in the limit of large parameters.

In the following, we extend the discussion to the *N*-edge-type case. We can show that an arbitrarily-chosen *N*-edge-type (*N* ∈ [1, *K*]) nested SC-LDPC code ensemble 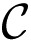
_Φ_ = {*d_v_*__Φ__, *d_c_*__Φ__, *M*, *L*, *w*} embedded in the MET code with node degrees {(*d_v_l__* ,*d_c_l__*) : *l* ∈ Φ} has the same design rate and the same BP threshold as a standard SC-LDPC code {Σ*_l_*_∈Φ_ *d_v_l__*,*d_c_*, *M*, *L*, *w*} if *d_c_l__* = *d_c_* for all *l* ∈ Φ. The proof can be done recursively as follows. We choose arbitrarily a two-edge-type nested SC-LDPC code 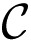
{*m,n*} = {*d_v_*_{_*_m_*_,_*_n_*_}_*,d_c_*_{_*_m_*_,_*_n_*_}_, *M*, *L*,*w*} from the MET code, where ***m*** ∈ Φ, *n* ∈ Φ and *m ≠ n*. This code is equivalent to a standard SC-LDPC code {*d_v_m__* + *d_v_n__*, *d_c_*, *M*, *L*, *w*} when *d_c_m__* = *d_c_n__* = *d_c_m__*threshold as a standard SC-LDPC code The edges of type-m and type-n can then be considered as the same type of edge. Now, we chose arbitrarily a type *l*, *l* ∈ Φ, *l* ≠ *m* and *l* ≠ *n* and add all type-*l* edges to the existing structure. If *d_c_l__* = *d_c_*, we obtain a code that has the same design rate and BP threshold as a standard SC-LDPC code {*d_v_m__ +d_v_n__ +d_v_l__*, *d_c_*, *M*, *L*, *w*} and is therefore capacity achieving. We repeat the above procedure until all of the *N* types of edges are added to the graph. The order in which we added the new edge type to the graph does not affect the result.

Based on the above derivation, we obtain the conclusion: if *d_c_l__* = *d_c_*∀*l* ∈ [0,*K* − 1], all of the arbitrarily-chosen N-edge-type (*N* ∈ [1, *K*]) nested SC-LDPC code ensembles embedded in the MET code are simultaneously capacity achieving in the limit of large parameters.

## 6. MET Nested SC-LDPC Codes for the Multiway Relay Channel

In this section, we apply the MET nested SC-LDPC codes for the multiway relay channel with full data exchange. We will prove that the capacity region of the model can be achieved by using the proposed code structure.

Again, we start with the case of two users and one relay. When applying the spatially-coupled LDPC codes, the connections between the variable nodes and the three types of check nodes are characterized by {*d_v_*__0__, *d_c_*__0__, *M*, *L*, *w*}, {*d_v_*__1__, *d_c_*__1__, *M*, *L*, *w*} and {*d_v_*__2__, *d_c_*__2__, *M*, *L*, *w*}. We denote the MET nested SC-LDPC code ensemble by 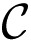 = {*d_v_*_{0,1,2}_*,d_c_*_{0,1,2}_, *M*, *L*, *w*}. The embedded two-edge-type SC-LDPC code, which is to be decoded at *U*_1_, is denoted by 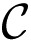
_{0,1}_ = {*d_v_*_{0,1}_ ,*d_c_*_{0,1}_, *M*, *L*, *w*}. Similarly, the two-edge-type code to be decoded at *U_2_* is denoted by 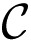
_{0,2}_ = {*d_v_*_{0,2}_, *d_c_*_{0,2}_, *M*, *L*, *w*}. If we write the MET SC-LDPC codes in the form of parity-check matrix, the only difference to Equation (3) is that the parity-check matrices are now for SC-LDPC codes instead of LDPC codes.

We apply the theoretical results in Section 5.3 for the performance analysis. Following Section 5.3, the two-edge-type codes 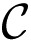
_{0,1}_ and 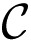
_{0,2}_ are simultaneously capacity achieving. Based on these facts, we give the following theorem.

**Theorem 4.** *The capacity region of the two-way broadcast channel with a common message can be achieved by using an MET nested SC-LDPC code* 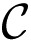 = {*d_v_*_{0,1,2}_, *d_c_*_{o,1,2}_, *M*, *L*, *w*}.

**Proof.** We assume that the capacities of the two individual links are *C*_1_ and *C*_2_, which together form the bound of the capacity region. The rate for broadcasting ***m***_0_ is assumed to be *R*_0_, and *R*_0_ ≤ min(*C*_1_,*C*_2_). For constructing an MET nested SC-LDPC code, we first choose a check degree *d_c_* which is the same for all types of check nodes. With this *d_c_*, we decide the variable degrees by satisfying:

$$\begin{array}{r} 1-\frac{d_{v_{0}}+d_{v_{1}}}{d_{c}}=C_{1}\\ 1-\frac{d_{v_{0}}+d_{v_{2}}}{d_{c}}=C_{2}\\ 1-\frac{d_{v_{0}}+d_{v_{1}}+d_{v_{2}}}{d_{c}}=R_{0} \end{array}$$

After determining the parameters, we construct an MET nested SC-LDPC code 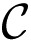 = {*d_v_*_{0,1,2}_, *d_c_*_{0,1,2}_, *M*, *L*, *w*} as explained in Section 5.2.

Assume that the numbers of three types of check nodes are *N_C_*__0__, *N_C_*__1__ and *N_C_*__2__, respectively. The number of variable nodes is *N_v_*. According to the definition of encoding rate in Equations (4)–(6), we have *R*_0_ = 1 − (*N_C_*__0__ + *N_C_*__1__ + *N_C_*__1__)/*N_v_*, *R*_1_ = *N_C_*__1__/*N_V_*, and *R*_2_ = *N_C_*__2__/*N_V_*. The design rate of the code 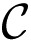
_{0,1}_ for *U*_1_ is given as:

$$R(d_{v\{0,1\}},d_{c\{0,1\}},M,L,w)=1-\frac{N_{C_{0}}+N_{C_{1}}}{N_{V}}=R_{0}+R_{2}$$

Since the code 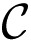
_{o,1}_ is capacity achieving, *i.e.*, in the limit of large *M*, *L* and *w*,

$$R(d_{v\{0,1\}},d_{c\{0,1\}},M,L,w)=1-\frac{d_{v_{0}}+d_{v_{1}}}{d_{c}}=C_{1}$$

it is easy to see that:

$$R_{0}+R_{2}=C_{1}$$

In a similar manner, for the relay- *U*_2_ link, we can prove that:

$$R_{0}+R_{1}=C_{2}$$

Therefore, the capacity region can be achieved by using the MET nested SC-LDPC code.

Now, we generalize to the multiway relay case. When constructing the MET nested SC-LDPC code, the connection between the variable nodes and the *l*-th type of check nodes is realized by the individual code {*d_v_l__*, *d_c_l__*, *M*, *L*, *w*}, *l* ∈ [0, *K* − 1]. In the following, we briefly discuss the performance of the proposed code construction in the multiway relay channel.

**Theorem 5.** *The capacity region of the multi-user broadcast channel with full data exchange can be achieved by using multi-edge-type SC-LDPC codes*.

**Proof.** For the given channel capacities {C*_l_* : *l* ∈ [1, *K* − 1]} and the rate *R*_0_, we choose the node degrees by satisfying:

$$1-\frac{\sum_{l=0}^{K-1}d_{v_{l}}}{d_{c}}=R_{0}$$

and:

$$1(d_{v_{0}}+\sum_{i:m_{i}\in S_{l}}d_{v_{i}})/d_{c}=C_{l}$$

for *l* ∈ [1, *K* − 1]. For a predetermined check degree *d_c_*, there must exist a unique nonnegative solution set {*d_v_*__0__, *d_v_l__*, …, *d_v_l__*,…, *d_v_*__K−1__} satisfying the *K* independent equations on condition that *R*_0_ ≤ min(*C*_1_,*C*_2_,…,C*_K_*_−1_)and *C_l_* ≥ *C_k_* if 
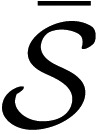
*_l_* ⊆ 
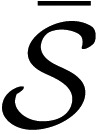
*_k_* for *l, k*, ∈ {1, 2, …, *K* − 1}.

We have shown in Section 5.3 that all of the N-edge-type nested SC-LDPC codes are simultaneously capacity achieving in the limit of large parameters. Therefore, the rate to be decoded at *U_l_* is asymptotically:

$$R_{0}+\sum_{i:m_{i}\in {\bar{S}}_{l}}R_{i}=1-(d_{v_{0}}+\sum_{i:m_{i}\in S_{l}}d_{v_{i}})/d_{c}=C_{l}$$

for *l* ∈ [1, *K* − 1]. Then, the boundary of the capacity region in Theorem 1 is achieved.

## 7. Numerical Results

In this section, we present the numerical results in terms of BP decoding thresholds and bit erasure rates (BERs) for the proposed MET nested SC-LDPC codes. The results are based on the density evolution for finite node degrees and based on simulations with finite code lengths. Without loss of generality, we only include the results for the two-way relay channel. The results for the multiway relay channel present the same characteristics, and therefore, they are omitted here.

Since we have shown in Section 5.3 that both the two-edge-type nested SC-LDPC codes 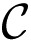
_{0,1}_ and 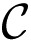
_{0,2}_ are capacity achieving, we consider only 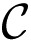
_{0,1}_ for the performance evaluation. We set the parameters as *d_V_*__0__ = 3, *d_c_* = 10, *w* = 3 and *L* = 100. Different variable degrees *d_V_*__1__ ∈ {1,2, 3, 4, 5, 6} are used to realize different sum rates *R*_0_ + *R*_2_, where the tradeoff between *R*_0_ and *R*_2_ is controlled by *d_V_*__2__, which is upper bounded by *d_V_*__2__ *≤ d_c_* − (*d_V_*__0__ + *d_v_*__1__) (see also Equations (4)–(6)). The results over BECs are summarized in Table 1. For the purpose of comparison, we also provide in Table 2 the results for regular LDPC block codes with the same node degrees. For each given degree setup, we calculated the gap between the Shannon limit and the BP threshold, which indicates how far the achievable rate at *U*_1_ is from the boundary of the capacity region.

We can see that the two-edge-type code 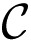
_{o,1}_, which is decoded at *U*_1_, exhibits significantly smaller gaps to capacity compared with its block code counterpart. In particular, regular LDPC block codes suffer greatly from larger gaps for lower rates. We observe a similar phenomenon for SC-LDPC codes, but the gaps are in general much smaller. In addition, the gaps can be further narrowed if we increase *L* for a given *w* or if we increase *w* while keeping the design rates fixed by choosing proper *L*. When both *w* and *L* are sufficiently large, the gaps for all degree distributions tend to disappear.

Similarly, we give the BP thresholds of SC-LDPC codes and regular LDPC block codes for BI-AWGN channels. The parameters are the same as in the BEC case, and the results are summarized in Tables 3 and 4. As for the BECs, the SC-LDPC codes clearly outperform the regular LDPC block codes in terms of the gap to the capacity

**Figure 4:**
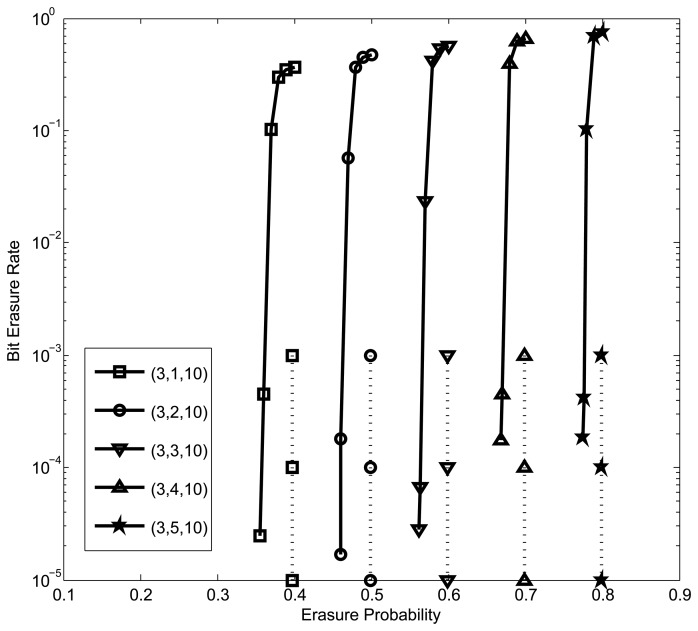
Bit erasure rates of the multi-edge-type (MET) spatially-coupled (SC)-LDPC codes over BECs. The solid curves show the simulation results, and the dotted vertical lines indicate the BP thresholds from density evolution.

In the following, we provide the simulation results for the SC-LDPC codes with finite code lengths over BECs. The bit erasure rates are plotted in Figure 4 together with the BP thresholds of the corresponding ensembles. The node degrees (*d_V_*__0__, *d_V_*__1__, *d_c_*) are listed in the legend, and we set *M* = 1000 and *L* = 100. It can be seen that the MET SC-LDPC codes generally provide good performance over BECs. Similar observations can be obtained in BI-AWGN channels, and the results are omitted here. We also want to point out that the complexity and latency due to the encoding and decoding of SC-LDPC codes are not an issue for their applications in practice, since SC-LDPC codes allow recursive encoding and sliding-window decoding.

## 8. Conclusions

In this manuscript, we proposed multi-edge-type spatially-coupled LDPC codes through coset encoding for the multiway relay channel with full data exchange. This model has various applications in wireless sensor networks, and we have provided a practical coding solution for the communication aspects of the model. We have proven that an arbitrarily-chosen *N*-edge-type SC-LDPC code embedded in the multi-edge-type construction is capacity achieving. By applying the above code construction to the broadcast phase of the multiway relay channel, each user recovers the message from the relay and all of the other users based on the receiver side information. We have proven that the capacity region of the specific model can be achieved by using the proposed construction. Numerical results were provided in both binary erasure channels and AWGN channels, which verified the theoretical analysis.

## Figures and Tables

**Table 1:** Belief-propagation (BP) thresholds of 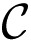
_{0,1}_ over binary erasure channels (BECs).

**(*d****_v_***__0__, *d****_v_***__1__)**	**Design Rate**	**Shannon Limit *ϵ*^Sh^**	**BP Threshold *ϵ*^BP^**	**Gap**
(3,1)	0.5921	0.4079	0.3970	0.0109
(3,2)	0.4902	0.5098	0.4989	0.0109
(3,3)	0.3882	0.6118	0.5961	0.0157
(3,4)	0.2862	0.7138	0.6861	0.0277
(3,5)	0.1883	0.8157	0.7654	0.0503
(3,6)	0.0823	0.9177	0.8328	0.0849

**Table 2:** BP thresholds of LDPC block codes over BECs.

**(*d****_v_***__0__, *d****_v_***__1__)**	**Design Rate**	**Shannon Limit *ϵ*^Sh^**	**BP Threshold *ϵ*^BP^**	**Gap**
(3,1)	0.6	0.4	0.3079	0.0921
(3,2)	0.5	0.5	0.3415	0.1585
(3,3)	0.4	0.6	0.3656	0.2344
(3,4)	0.3	0.7	0.3840	0.3160
(3,5)	0.2	0.8	0.3990	0.4010
(3,6)	0.1	0.9	0.4115	0.4885

**Table 3:** BP thresholds of 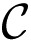
_{0,1}_ over binary-input (BI)-AWGN channels.

**(*d****_v_***__0__, *d****_v_***__1__)**	**Design Rate**	**Shannon Limit *ϵ*^Sh^**	**BP Threshold *ϵ*^BP^**	**Gap**
(3,1)	0.5921	0.854	0.836	0.018
(3,2)	0.4902	0.994	0.976	0.018
(3,3)	0.3882	1.173	1.143	0.030
(3,4)	0.2862	1.427	1.355	0.072
(3,5)	0.1883	1.829	1.630	0.199
(3,6)	0.0823	2.751	1.998	0.753

**Table 4:** BP thresholds of LDPC block codes over BI-AWGN channels.

**(*d****_v_***__0__, *d****_v_***__1__)**	**Design Rate**	**Shannon Limit ∈^Sh^**	**BP Threshold ∈^BP^**	**Gap**
(3,1)	0.6	0.844	0.748	0.096
(3,2)	0.5	0.979	0.793	0.186
(3,3)	0.4	1.149	0.827	0.322
(3,4)	0.3	1.386	0.853	0.533
(3,5)	0.2	1.767	0.875	0.892
(3,6)	0.1	2.552	0.894	1.658
